# Leading from the Centre: A Comprehensive Examination of the Relationship between Central Playing Positions and Leadership in Sport

**DOI:** 10.1371/journal.pone.0168150

**Published:** 2016-12-15

**Authors:** Katrien Fransen, S. Alexander Haslam, Cliff J. Mallett, Niklas K. Steffens, Kim Peters, Filip Boen

**Affiliations:** 1 Department of Kinesiology, KU Leuven, Leuven, Belgium; 2 School of Psychology, The University of Queensland, St. Lucia, Queensland, Australia; 3 School of Human Movement and Nutrition Sciences, The University of Queensland, St. Lucia, Queensland, Australia; Tianjin University of Technology, CHINA

## Abstract

**Research aims:**

The present article provides a comprehensive examination of the relationship between playing position and leadership in sport. More particularly, it explores links between leadership and a player’s *interactional centrality*—defined as the degree to which their playing position provides opportunities for interaction with other team members. This article examines this relationship across different leadership roles, team sex, and performance levels.

**Results:**

Study 1 (*N* = 4443) shows that athlete leaders (and the task and motivational leader in particular) are more likely than other team members to occupy interactionally central positions in a team. Players with high interactional centrality were also perceived to be *better* leaders than those with low interactional centrality. Study 2 (*N* = 308) established this link for leadership in general, while Study 3 (*N* = 267) and Study 4 (*N* = 776) revealed that the same was true for task, motivational, and external leadership. This relationship is attenuated in sports where an interactionally central position confers limited interactional advantages. In other words, the observed patterns were strongest in sports that are played on a large field with relatively fixed positions (e.g., soccer), while being weaker in sports that are played on a smaller field where players switch positions dynamically (e.g., basketball, ice hockey). Beyond this, the pattern is broadly consistent across different sports, different sexes, and different levels of skill.

**Conclusions:**

The observed patterns are consistent with the idea that positions that are interactionally central afford players greater opportunities to *do* leadership—either through communication or through action. Significantly too, they also provide a basis for them to *be seen to do* leadership by others on their team. Thus while it is often stated that “leadership is an action, not a position,” it is nevertheless the case that, when it comes to performing that action, some positions are more advantageous than others.

## Introduction

The presidential speechwriter James Humes once observed that “the art of communication is the language of leadership” [1]. To increase access to their audience, it is therefore common for powerful people to occupy a conspicuous position in their group. Political leaders are placed on a podium, teachers are positioned in the front of a class, and managers are seated at the head of the table. All these leaders seek out these prominent positions with the aim of maximizing their visibility and increasing their influence over other group members. Because these positions allow leaders to engage with team members, we will refer to them as *interactionally central positions* (i.e., comprising a high degree of *interactional centrality*). In any social context, the most interactionally central position is the one that affords the greatest potential to interact with other group members (e.g., an audience, pupils, or employees). This construct will often be highly correlated with, but nevertheless differs from, what we can refer to as *spatially central positions*— in which a person is simply physically close to other group members (i.e., a central position on a playing field [2]). In a theatre, for example, the actors on stage will typically be interactionally but not spatially central.

Organizational research studying communication networks (e.g., [3, 4]) has demonstrated that members who occupy interactionally central positions in their organization also tend to emerge as leaders (for reviews, see [5, 6]). A key reason for this is that these members are in a better position to communicate with other team members. Interactional centrality puts them in a better position to control the flow of information and to coordinate the group’s activities. Indeed, on the basis of a review of relevant research, Grusky [7] concludes that “all else being equal, the more (*interactionally*) central one’s location: (1) the greater the likelihood coordinative tasks will be performed, and (2) the greater the rate of interaction with the occupants of other positions.”

Effective communication is vital, not only in organizations, but also in sports teams. Coaches, for example, need to demonstrate high-quality communication skills in their interactions with players, assistant coaches, club management, and media [8]. Within the team communication is also central to team effectiveness [9, 10], and here athlete leaders (i.e., athletes who occupy a leadership role) have a key role in optimizing communication flow within the team (for a review on athlete leadership, see [11]). Accordingly, an interactionally central position should benefit athlete leaders by facilitating their communication with other team members (both verbally and non-verbally). Furthermore, a central position of this form should allow athlete leaders to have more influence on the game than a peripheral position.

In line with this logic, several studies of sports teams have revealed a link between athlete leadership and interactional centrality. In particular, Lee, Patridge, et al. [12] found that professional soccer players who occupied an interactionally central playing position (i.e., as a midfielder or central defender) were more likely to be given the role of team captain. Melnick and Loy [13] corroborated these findings in rugby union—observing that 35.5% of team captains occupied the two most spatially central positions (i.e., the half back and Number 8), while there was not a single captain who held one of the three most peripheral spatial positions on the field. Furthermore, the authors noted that these positions were not only spatially central (i.e., centrally located on the field), but also interactionally central in so far as they constituted the crucial link between the forwards and the backs. Similar patterns have also been observed in baseball, where more effective leaders (based on coach ratings) tend to occupy more interactionally central field positions [14].

However, not all studies have observed this link between athlete leadership and interactional centrality. For example, Tropp and Landers [15] found that captains in female field hockey teams were less likely to play in an interactionally central position. They also found that goalies (i.e., the least interactionally central position) received the best leadership ratings. In ice hockey too no link was observed between leadership and interactional centrality [16]. However, because the sports in which these studies were conducted vary on a range of dimensions, this discrepancy might be attributable to a number of factors specific to the different contexts (e.g., the nature of the sport, the level at which it was being played, players’ sex).

### The Present Research

The study reported below attempts to address the relationship between leadership and interactional centrality and to resolve some of the inconsistencies between findings identified in the foregoing review. It does so by addressing four limitations that are inherent to the extant literature.

First, all previous studies have relied on a very specific sample. As a result, they are unable to shed light on the extent to which the importance of interactional centrality for leadership is contingent upon features of a particular sport (e.g., rugby vs. field/ice hockey), the sex of the players, or on the performance level (e.g., professional versus recreational). More specifically, it is likely that sport-specific characteristics such as the number of players, the size and shape of the field, position specificity, and the extent to which players switch positions during the game impact upon the distribution of interactional centrality within a team. For example, in soccer (in which there are many players, a large field, and relatively fixed positions) there are considerable differences in the opportunities for communication that are available to players in central and non-central positions (e.g., to a midfielder vs. a goal keeper).Yet in other sports, such as basketball (where there are a small number of players, who dynamically switch positions during a game, and where the court is relatively small), it is easy for all players to interact with their teammates. As a result, there is less variance in the interactional centrality of different team members.

By collecting samples from multiple sports, male and female teams, and different performance levels, the present research aims to gain more insight into the role that these various features play in the relationship between athlete leadership and interactional centrality. Given that group processes are assumed to be no different for male and female sport participants, we expect support for our hypotheses in both male and female teams. Relatedly, since sport-specific field positions and associated regulations do not differ across performance levels (e.g., national, regional, or youth), we also expect generalized support for our hypotheses across performance levels. However, on the basis of the above reasoning, we hypothesize that:

H1: Different sports will be associated with more or less variability in interactional centrality and this will be associated with stronger or weaker relationships between interactional centrality and leadership. More specifically, we hypothesize that the link between interactional centrality and leadership will be stronger to the extent that there is high variability in interactional centrality of the different players because the sport is played (a) in a large area, (b) with more players, and (c) with players occupying relatively fixed positions.

This means, for example, that the relationship between interactional centrality and leadership should be stronger in soccer, rugby, field hockey and water polo than in basketball, handball, volleyball and ice hockey.

A second limitation of previous research is that it has tended to equate leadership with the role of team captain and hence focused on the interactional centrality of those who occupy this role. However, recent research points to the fact that team captains are not always perceived to be the best leaders of their team. Instead, it seems that most leadership roles are occupied by informal leaders [17]. Accordingly, in the present research, we are interested not only in the position of formal leaders (e.g., team captains), but also in the position of informal leaders. To this end, we will use social network analysis to identify the leadership quality of *all* players in a team (based on the perceptions of all other team members) regardless of their leader status.

A third limitation of previous work is that it has been concerned with leadership a single unitary construct. However, recent research has observed that there is a range of different ways in which athletes can perform leadership with a team. In particular, Fransen, Vanbeselaere, et al. [17] distinguish between four leadership roles that athletes can occupy both formally and informally: the task and motivational leader on the field and the social and external leader off the field (for full definitions, see Table 1).

**Table 1:** Four distinct leadership roles in sport (as defined by Fransen, Vanbeselaere, et al., 2014).

Leadership role	Definition
Task leader	A task leader is in charge on the field; this person helps the team to focus on our goals and helps in tactical decision-making. Furthermore the task leader gives his/her teammates tactical advice during the game and adjusts them if necessary.
Motivational leader	The motivational leader is the biggest motivator on the field; this person can encourage his/her teammates to go to any extreme; this leader also puts fresh heart into players who are discouraged. In short, this leader steers all the emotions on the field in the right direction in order to perform optimally as a team.
Social leader	The social leader has a leading role besides the field; this person promotes good relations within the team and cares for a good team atmosphere, e.g. in the dressing room, in the cafeteria or on social team activities. Furthermore, this leader helps to deal with conflicts between teammates besides the field. He/she is a good listener and is trusted by his/her teammates.
External leader	The external leader is the link between our team and the people outside; this leader is the representative of our team toward the club management. If communication is needed with media or sponsors, this person will take the lead. This leader will also communicate the guidelines of the club management to the team regarding club activities for sponsoring.

Although a particular player could theoretically occupy more than one of these leadership roles, previous research indicates that these different leadership roles are often fulfilled by different members of a team [17, 18]. The present research will therefore investigate the relationship between interactional centrality and athlete leadership not only for leadership in general, but also for each of these four distinct roles. This will allow us to compare the importance of interactional centrality for on-field and off-field leaders. Moreover, the fact that the leadership function of the social and external leader is enacted off the field, we hypothesize that:

H2: Interactional centrality will be related more strongly to task and motivational leadership than to social and external leadership (H2a). Furthermore, given that team captains tend to occupy on-field rather than off-field leadership roles [17], team captains will be more likely than other players to occupy interactionally central field positions (H2b).

A final shortcoming of previous research in this area is that it has focused on players’ occupation of a leadership role (i.e., as team captain), rather than on the quality of their leadership. However, having a formal leadership position is not always a good proxy for *leadership quality*. Accordingly, we will investigate whether having an interactionally central position is also linked to player’s perceived leadership quality. For this purpose, we will use social network analysis to establish the perceived leadership quality of every player in a team, as rated by all other team members. On this basis, we can examine whether players with interactionally central playing positions are perceived to be better leaders than those who occupy non-central positions, regardless of their formal leadership status. Moreover, given that an interactionally central field position relates to leadership on rather than off the field, we hypothesize that:

H3: Interactional centrality will generally be a better predictor of the perceived quality of task and motivational leaders than of the perceived quality of social and external leaders.

## Methods

To examine the above four hypotheses, we conducted four different studies. In Study 1, we investigated the first research question (i.e., are leaders more likely than their teammates to play in a central position?) by asking players and coaches in nine different sports to complete an online questionnaire. Studies 2, 3, and 4 were designed to answer our second research question (i.e., is interactional centrality related to leadership *quality*?). Here, the recruitment of complete teams (in contrast to the individual player recruitment in Study 1) allowed us to conduct social network analysis to map the leadership qualities of the whole team and link them to particular playing positions. While we investigated general leadership quality in Study 2, we went more into detail in Study 3 and Study 4 by relating playing positions to the leadership quality of players in each of the four leadership roles (i.e., task, motivational, social, and external leader). Study 4 was designed to replicate Study 3 in a different and larger sample.

### Procedures

All studies used different samples to examine their research question. In Study 1, 8,509 players and 7,977 coaches were invited via e-mail to complete an online questionnaire; 3,193 players and 1,258 coaches replied, resulting in an estimated total response rate of 27% (i.e. 37.5% for players and 15.8% for coaches). In Study 2, 40 teams were invited via e-mail to participate, and 35 teams accepted this invitation (a response rate of 88%). In Study 3, a similar procedure led to the participation of 24 sport teams (a response rate of 77%). For Study 4, we invited 130 sport teams via e-mail, resulting in 64 teams which agreed to participate (response rate of 49%).

In Studies 2, 3, and 4, a research assistant attended a training session of the participating teams, where a paper-and-pencil survey method was administered. The design of the different studies was approved by the ethics committee of KU Leuven, Belgium. Informed consent was obtained from all participants, no rewards were given, and full confidentiality was guaranteed. Data from these samples, which were part of a larger project, have been published in other research articles (e.g., [17–20]), but these related to very different research questions (in particular, playing position was not a variable of interest).

### Participants

#### Study 1

The sample included 4,333 participants, who were active in eight different sports (basketball, volleyball, soccer, handball, field hockey, rugby, water polo, and ice hockey). The original data collection also included 118 korfball participants. However, because korfball is a mixed-gender sport without specific positions (i.e., each player can play every position on the court), we omitted these data from our analysis. Demographic details of all participants are presented in Table 2. In this study (and those below) we focus on the four sports that are most often played in the country where the research was conducted (Belgium)—namely soccer, basketball, volleyball, and handball.

**Table 2:** Demographics of the participants.

	Study 1		Study 2	Study 3	Study 4
	Players	Coaches	Players	Players	Players
*N*	3,108	1,225	308	267	776
Sex					
Male	1,876 (40%)	1,106 (90%)	188 (61%)	140 (52%)	380 (49%)
Female	1,232 (40%)	119 (10%)	120 (39%)	127 (48%)	396 (51%)
Age	23.9	41.9	24.9	24.3	23.8
	(*SD* = 7.1)	(*SD* = 12.1)	(*SD* = 7.5)	(*SD* = 4.9)	(*SD* = 6.3)
Years of playing/coaching experience	14.1	14.0	15.7	14.9	14.7
	(*SD* = 14.2)	(*SD* = 10.2)	(*SD* = 7.0)	(*SD* = 5.8)	(*SD* = 6.7)
Team tenure	5.8	2.6	6.5	3.7	5.0
	(*SD* = 5.5)	(*SD* = 2.9)	(*SD* = 7.2)	(*SD* = 3.4)	(*SD* = 4.9)
Competitive level					
High level (i.e., national level)	949 (31%)	335 (27%)	175 (57%)	149 (56%)	428 (55%)
Low level (i.e., provincial, regional, and recreational level)	2,054 (66%)	737 (60%)	133 (43%)	118 (44%)	348 (45%)
Youth level	105 (3%)	153 (13%)	/	/	/
*N*_Sport_					
Basketball	1,551 (50%)	408 (33%)	63 (21%)	77 (29%)	134 (17%)
Soccer	249 (8%)	340 (28%)	100 (33%)	97 (36%)	247 (32%)
Volleyball	919 (30%)	368 (30%)	75 (24%)	93 (35%)	161 (21%)
Handball	76 (2%)	40 (3%)	70 (23%)	/	234 (30%)
Hockey	110 (4%)	17 (1%)	/	/	/
Ice hockey	59 (2%)	13 (1%)	/	/	/
Rugby	60 (2%)	24 (2%)	/	/	/
Water polo	84 (3%)	15 (1%)	/	/	/

#### Study 2

Thirty-five sports teams participated in this study (8 volleyball teams, 8 soccer teams, 8 basketball teams, and 11 handball teams). To conduct reliable social network analyses, a high response rate within each participating team is required [21, 22]. Accordingly, we removed 10 teams that did not have a response rate of at least 75% [23]. The demographics of the 25 remaining teams are presented in Table 2. By using a stratified sampling technique, we ensured that our sample consisted of equal numbers of male and female teams, and of teams playing at either a high level (i.e., national level) or at a low level (provincial or regional level).

#### Study 3

Twenty-four sports teams participated in this study (8 soccer teams, 8 volleyball teams, and 8 basketball teams). Based on the response rate cut-off of 75% for each team, 3 teams were removed from the dataset. Descriptive statistics for the remaining sample of 21 teams can be found in Table 2. As in Study 2, we adopted a stratified sampling technique to ensure an equal number of teams of each sex (i.e., male/female) and performance level (i.e., high/low level).

#### Study 4

This study included 64 sports teams (i.e., 16 soccer teams, 16 volleyball teams, 16 basketball teams, and 16 handball teams). The response rate of all of these teams exceeded the cut-off of 75%. As in the previous study, we adopted a stratified sampling technique to ensure an equal number of teams of different sex and performance level. In the three latter studies, the same criteria were used to differ between teams playing at either high level (i.e., national level) or low level (provincial or regional level). Demographics of all participants can be found in Table 2.

### Leadership Assessment

#### Study 1

To identify the different leaders in each team (i.e., task, motivational, social, and external leader), participants were asked to read definitions of each of these leadership roles (see Table 1; based on [17]). Next, they indicated the name of the player in their team who best fulfilled each of the four leadership roles. Participants could only indicate one player for each role, but it was possible for the same player to be identified in different leadership roles. Leaders did not need to have formal status as a leader (e.g., as team captain) in order to be identified as such.

#### Study 2

This study focused on leadership *quality* not just leader appointment. Because the study included complete teams, social network analysis could be used to create a leadership quality score for each player based on the perceptions of all team members. For this purpose, each participant had to indicate “to what extent they considered each player as having good general leadership qualities” on a 5-point Likert scale, ranging from 0 (*very poor leader*) to 4 (*very good leader*). To standardize the procedure, all the names of the players on the team were listed in advance. Based on all the responses in the team, a social network was created, in which the nodes represented the different players in the team and the directed relations between the nodes referred to the rating of general leadership quality that team members gave each other.

To assess the leadership quality of a given player, we used the social network measure of indegree centrality, which represents the average of players’ perceptions of leadership quality. As suggested by previous research, this measure represents a leader’s importance in the network and his/her influence over other team members [24–26]. The best leader for each leadership role (i.e., task, motivational, social, or external leader) was identified as the player with the highest leadership indegree centrality (i.e., the average of all the received leadership ratings of team members).

#### Study 3

Instead of assessing team members’ perceptions of their teammates’ general leadership quality, in this study players were asked to rate the quality of each team member as (a) task leader, (b) motivational leader, (c) social leader, and (d) external leader on 5-point Likert scales, ranging from 0 (*very poor leader*) to 4 (*very good leader*). This procedure resulted in the identification of four different leadership networks for each team. For each leadership role, we calculated a leadership score for each player by identifying the player’s indegree centrality in that particular leadership network (i.e., the average of all the received leadership ratings of team members in a particular leader role).

#### Study 4

As in Study 3, this study assessed perceptions of players’ leadership quality in the four different leadership roles. However, in light of the fact that leadership ratings in Study 3 tended to be in the upper half of the scale, here we used 7-point scales that provided opportunity for more nuanced ratings (where 1 = *very poor leader*, 7 = *very good leader*).

### Playing Positions

In Study 1 we asked participants to indicate the playing position of the leader they identified in each leadership role, as well as the playing position of the team captain. Answers on these open-ended questions were thereafter recoded for each particular sport in the same categories as in the other studies. In Studies 2, 3 and 4, participants were also asked to indicate their own playing position on a predefined list of the different playing positions in their particular sport.

### Interactional Centrality

As outlined in the Introduction, centrality in this article refers to the *interactional* centrality of a position rather than its *spatial* centrality. To categorize team positions as interactionally central or peripheral in volleyball, basketball, and soccer, we relied on the work of Clemente and colleagues [27–29], who constructed networks in which the ties represented the passes between the team members and where team members who were positioned most centrally in the network demonstrated the highest interaction with their team members.

Using this procedure in volleyball, it was shown that the setter was the most prominent person in receiving the ball and passing it to other players [27]. Given the classic three-pass structure in volleyball, in which the setter aims to have the second contact, this should come as no surprise. The same procedure revealed that in basketball the point guard occupied the most central position, followed by the shooting guard [28]. Because no significant difference was observed in the centrality metrics between these two positions, both guard positions were treated as central positions. Furthermore, in soccer, the (left, central, and right) midfielders were the prominent players followed by the central defenders [30]. Again, because no significant differences emerged between the centrality metrics of the two positions, we defined both the midfielder positions and the central defender position as central positions. This categorization is in line with previous research by Lee, Patridge, and Coburn [12] which identified both midfielders and central defenders as occupying central playing positions.

In field hockey, interaction analysis revealed that midfielders (i.e., the inside-left and inside-right (inners) positions) had the most interaction with team members [15]. In ice hockey, Roy [16] found that the players who interacted most with other team members occupied the center position. In rugby, Melnick and Loy [13] identified the half back (i.e., scrum-half and fly-half) and Number 8 positions as the most central positions.

As no research evidence was available for the two remaining sports (handball and water polo), we asked three experts in each of these sports to identify the position in their sport that was characterized by the most interaction with other team members (in terms of receiving/giving passes). Results indicated in handball ‘the center backcourt’ was seen to be that the most central position and that in water polo the central position was seen to be ‘the point’.

### Data Analysis

The analyses for Study 2, 3, and 4 are relatively straightforward; however, those for Study 1 are more complex. Here, to identify whether athlete leaders have a higher chance of playing in a central position than other team members, we compared the percentage of leaders in a central position to a reference percentage (i.e., the statistical likelihood of the leader occupying a central position, assuming a random distribution across the positions). The reference position was calculated by dividing the number of on-field central positions by the total number of on-field positions in that particular sport. Because the number of (central) positions varies according to each sport, the reference percentage is sport-specific. For example, in soccer we opted for the classic 4-4-2 playing system (i.e., three lines of players with respectively four, four, and two players on the same line), in which we defined both the midfielder positions (i.e., two on-field positions) and the central defender positions (i.e., two on-field positions) as central. Consequently, the reference percentage for soccer is $\frac{2+2}{11}=36.4\%$. For soccer, we also tested all our hypotheses with reference to a 4-2-3-1 system (i.e., four lines of players, including respectively four, two, three and one player) that included three midfielders and two central defenders (i.e., five central positions with a resultant reference percentage of 45.5%). However, our conclusions for this system remained exactly the same.

For the percentage of leaders playing in a central position, we used the valid percentage (i.e., omitting the missing values and the respondents who assigned multiple positions to the leader). To identify whether athlete leaders have a higher chance of playing in a central position than other team members, for each sport we compared the valid percentages of athlete leaders playing in a central position with the reference percentage via a general *z*-test [31]. The significance level of this test is calculated via the following formula: $z=\frac{p-p_{exp}}{se\;(p)}$, where *p* is the observed proportion; *p*_exp_ is the null hypothesis proportion (in this case the reference percentage); and *se*(*p*) is the standard error of the reference percentage: $se\;(p)=\sqrt{\frac{p_{exp}(1-p_{exp})}{n}}$, with *n* being the valid sample size (i.e., total sample size of the sport, minus the missing values, minus the participants who assigned multiple positions to their leader).

Furthermore, when differences arose between male and female leaders or between the leaders at different performance levels, we compared the specific percentages of leaders playing at a central position within each of the categories using an *N–* 1 Chi-squared test, as recommended by Campbell [32] and Richardson [33].

## Results

### Interactional Centrality of Athlete Leaders

In Study 1, we asked participants to indicate the positions of their athlete leaders. The overview in S1 Table presents the playing positions of the team captain and the identified leaders in the four roles in eight different sports (with the central positions in each sport indicated by an asterisk). To identify whether leaders have a higher chance of playing in a central position than their fellow team members, we compared the percentage of leaders in a central position to the reference percentage (as defined above). Table 3 presents the valid percentages of leaders playing in a central position and the reference percentage for their sport. When comparing the different leadership roles, we see that in all sports except field hockey the percentage of leaders playing in a central position is highest for task leaders. In other words, the link between leader status and interactional centrality is strongest for task leadership.

**Table 3:** The valid percentage of athlete leaders playing in a central playing position across different sports (Study 1).

	Reference percentage	Task leader	Motivational leader	Social leader	External leader	Team captain
Total sample		48.9	30.6	25.6	29.3	35.2
Sport-specific						
Basketball	40.0	55.0***	25.6	20.6	27.5	38.9
Soccer	36.4	80.9***	67.0***	56.1***	66.9***	73.5***
Volleyball	14.3	27.6***	22.1***	22.4***	23.7***	18.2***
Handball	14.3	63.5***	12.7	13.3	7.9	23.2*
Hockey	27.3	39.8**	41.9**	27.5	38.2	41.9**
Ice hockey	16.7	27.6*	23.4	11.4	10.5	18.5
Rugby	20.0	43.3***	42.3***	19.4	20.7	42.6***
Water polo	14.3	52.5***	35.7***	33.3**	47.4***	51.0***

Note. Significance levels indicate the percentages that are significantly higher than the reference percentage for a given sport (i.e., the number of central on-field positions, divided by the total number of field positions for that sport). This percentage corresponds to the statistical likelihood of the leader occupying a central position, assuming a random distribution across the positions.

*p < .05

**p < .01

***p < .001

Furthermore, for each leadership role we compared whether leaders had a higher chance than other team members of playing in a central position. In line with H2a, results clearly demonstrated that, relative to other leadership roles, the task leader had the greatest likelihood of playing in a central position. This finding was consistent across the eight examined sports. For most sports H2a was also supported in the case of motivational leadership, where findings also revealed a significant link between motivational leader status and interactional centrality (i.e., in soccer, volleyball, field hockey, rugby, and water polo). In other words, in these five sports, motivational leaders were more likely than other team members to play in a central position. However, in basketball, handball, and ice hockey, no significant link between interactional centrality and motivational leadership emerged, nor with any of the off-field leadership roles. This differentiation across sports provides support for H1 as basketball, handball, and ice hockey are played on a relatively small field and involve continual switching of positions— meaning that all players have similar communication opportunities and that a central position is not required in order to display leadership.

Finally, we also studied the interactional centrality of team captains. In line with H2b, our findings revealed a strong link between team captaincy and interactional centrality. In all sports (except basketball and ice hockey), team captains were more likely to occupy a central playing position than other team members. Exceptions in the case of basketball and ice hockey again support H1 in demonstrating the sport-specificity of the link between position centrality and leadership.

#### Differences between male and female teams

After examining sport-specific findings, we also investigated the differences between male and female teams. S2 Table presents the percentage of male and female athlete leaders who occupy a central position for each of the examined sports. Because the number of females in some sports is too low to produce reliable results, we decided to omit the percentages of all groups containing less than 10 valid cases, in line with guidelines suggested by Cochran [34]. Overall, we can conclude that in most sports and for most leadership roles, there is considerable similarity between male and female leaders. For example, in soccer and volleyball, both male and female leaders in each of the examined leadership roles were more likely than their teammates to occupy a central position.

If differences did arise between males and females (e.g., male leaders had significant higher likelihood of occupying a central position, but female leaders did not), an *N*–1 Chi-square test was conducted to examine whether the male and female percentages were significantly different, regardless of the reference percentage. The results are presented in S2 Table. Notable differences between male and female teams emerged in field hockey where female motivational and external leaders were more likely to occupy a central position than male leaders. Furthermore, male team captains in volleyball and in rugby were more likely than their female counterparts to occupy a central field position. Overall, though, support for our hypotheses was consistent across male and female teams.

#### Differences across performance levels

Adopting a similar procedure as for team sex, S3 Table provides details of the consistency of our findings across different performance levels. Again, all percentages of groups containing less than 10 valid cases were omitted. Overall, a high consistency emerged across different performance levels. The only exceptions related to the interactional centrality of team captains in basketball and in volleyball. In basketball, it was more likely that team captains of youth teams would occupy a central position than team captains at a more senior level. In volleyball, team captains at a low level were more likely than youth team captains to occupy a central position. However, these exceptions aside, there was generally a high degree of consistency across the different levels, which again supports our hypotheses.

### Relation between Interactional Centrality and Leadership Quality

**Fig 1:**
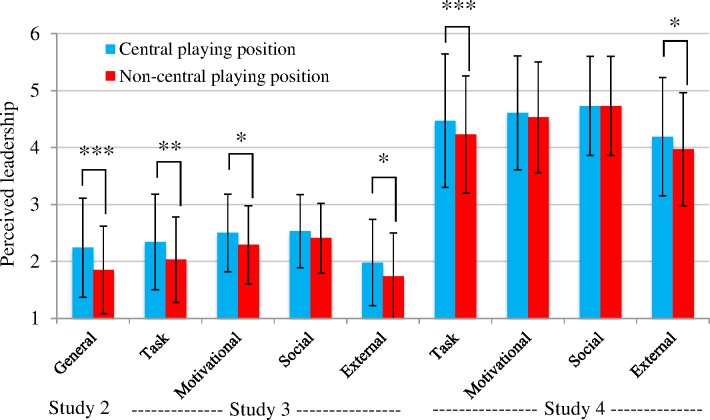
Mean perceived leadership quality of players occupying central and non-central positions (In Studies 2 and 3, leadership quality was rated from 0 to 4, in Study 4 it was rated from 1 to 7).

Having examined whether leaders are more likely to play in a central position than non-leaders, we wanted to examine whether interactional centrality is associated with perceived leadership quality. More specifically, we examined whether a player’s leadership quality is perceived to be higher if he or she plays in a central position. Study 1 only contained information about the leader and thus provided no useful information in this regard. However, because Studies 2, 3 and 4 assessed complete teams, here social network analysis allowed us to obtain a leadership rating for each player in the team. Accordingly, we were able to explore the association between the perceived leadership quality of all players and their interactional centrality. Speaking to this issue, Fig 1 presents the perceived leadership quality of players in central and non-central positions (for general leadership in Study 2 and each of the four leadership roles in Studies 3 and 4).

In **Study 2**, an independent sample *t*-test revealed that team members playing in central positions were on average generally perceived to be better leaders than those playing in other positions (*t* = 3.71; *p* < .001). This was the case in both male teams (*t* = 2.01; *p* < .05) and female teams (*t* = 3.58; *p* = .001); in both high-level teams (*t* = 2.46; *p* < .05) and low-level teams (*t* = 2.87; *p* < .01); and across sports—such that team members who played in a central position were perceived as better leaders in soccer (*t* = 2.15; *p* < .05), volleyball (*t* = 2.16; *p* < .05), and handball (*t* = 2.24; *p* < .05). Only in basketball were there no significant differences in perceived leadership quality as a function of interactional centrality.

**Study 3** revealed similar patterns in that players in central positions were perceived by their teammates as better task leaders (*t* = 2.86; *p* < .01), better motivational leaders (*t* = 2.29; *p* < .05), and better external leaders (*t* = 2.37; *p* < .05). However, the centrality of a player’s position was not associated with the quality of his/her perceived leadership in the social leadership role (*t* = 1.40; *p* = .16). These results largely confirm H3 in so far as interactional centrality seems to be a better predictor of on-field leadership quality than of off-field leadership quality (although the significant results for external leadership partly contradict this hypothesis).

Comparison of male and female teams in Study 3 indicated that in male teams players in central positions were perceived as better task leaders (*t* = 2.65; *p* < .01), better motivational leaders (*t* = 2.07; *p* < .05), better social leaders (*t* = 2.04; *p* < .05), and better external leaders (*t* = 2.62; *p* < .05). In female teams, however, no significant link emerged between perceived leadership quality and interactional centrality for any of these leadership roles.

Comparison of high- and low-level athletes indicated that at a high level, athletes in a central position are perceived as better task leaders (*t* = 2.47; *p* < .05), better motivational leaders (*t* = 2.43; *p* < .05), and better social leaders (*t* = 2.09; *p* < .05). There were no significant differences between athletes occupying central and non-central positions for external leadership or for teams playing at a low level.

When results are examined across different sports, there was no evidence of a link between perceived leadership quality and interactional centrality in volleyball and basketball. However, in soccer, players in central positions are perceived as better task leaders (*t* = 2.11; *p* < .05) and better motivational leaders (*t* = 2.26; *p* < .01) than their teammates in non-central positions. This pattern is in line with H1a. For social and external leadership, there were no significant differences in the leadership quality of soccer players who occupied central and non-central positions—a pattern that is consistent with H3.

**Study 4** replicated the findings of Study 3 for task and external leadership in so far as players in central positions were perceived by their teammates to be both better task leaders (*t* = 3.36; *p* = .001) and better external leaders (*t* = 2.35; *p* < .05). However, when it came to motivational and social leadership roles the centrality of players’ position was unrelated to their perceived leadership quality— indicating that here H3 was only partly confirmed.

In line with the findings of Study 3, associations between interactional centrality and leadership were again more evident in male than in female teams. However, when examining the results across performance levels (which are defined consistently across studies), the results contradicted those of Study 3. More specifically, at a high performance level there were no significant differences in the perceived leadership of players occupying central and non-central positions. By contrast, at a low performance level, players in a central position were perceived as significantly better task leaders (*t* = 4.37; *p* < .001), better motivational leaders (*t* = 2.60; *p* = .01), better social leaders (*t* = 2.34; *p* < .05) and better external leaders (*t* = 3.77; *p* < .001) than their teammates. In addition, no sport-specific differences were observed.

## Discussion

The present article provides a comprehensive examination of the relationship between athlete leadership and interactional centrality in sport. In four studies, we identified the playing positions not only of team captains but also of task, motivational, social, and external leaders (which can include both formal and informal athlete leaders). In addition, we investigated the link between the interactional centrality of leaders’ playing positions and their perceived leadership quality. So far as we know, the present research is the first that investigates these issues by combining large data sets that involve male and female teams, different sports, and different performance levels (high, low, and youth) that were defined consistently across the various studies. This variety in sample composition provided a unique opportunity to examine whether the importance of interactional centrality for athlete leadership varied as a function of these various factors.

### The Interactional Centrality of Athlete Leaders

When comparing the importance of interactional centrality across different roles, we see that in every sport the task leader was the player most likely to occupy a central position on the field. A likely explanation for this is that a central position affords task leaders the best opportunities to communicate with their fellow team members. As a result, task leaders should be ideally placed to communicate tactical strategies quickly throughout the team and to provide guidance to team members when necessary. Furthermore, the high level of interaction (in terms of ball contacts) that is inherent to central positions, allows the task leader to have more direct impact on the game itself.

In some sports, the identified central position also requires advanced tactical knowledge [35]. For example, in basketball, the guard controls the game and decides on the game strategy that is followed. In volleyball too, the setter plays the second contact and decides which player he/she passes the ball to (depending on the team’s strategy and that of the opponent). Likewise, in rugby the half backs and Number 8 control the distribution of the ball from the forwards to the backs and out to the different wings. In these sports, there is obviously a close link between the central position and the role of task leader, irrespective of sport-specific characteristics such as field size and position switching.

In most sports, motivational leaders also occupy an interactionally central position. However, this relationship did not emerge in basketball, handball, and ice hockey. The sport-specificity of this pattern can be explained by the fact that players in these three sports—all of which are characterized by regular switching of positions during play—all players have an equal opportunity to communicate with other team members. In line with Hypothesis 1, it therefore follows that in these sports players do not have to occupy central positions in order to motivate their fellow team members.

In line with Hypothesis 2a, interactional centrality was less important for off-field leadership roles. Nevertheless, in some sports (specifically, soccer, volleyball, and water polo) off-field leaders were also more likely to occupy interactionally central positions. This speaks to the fact that players can fulfil more than one leadership role at the same time—and that there is some ‘spill-over’ from one leadership role to another. Task and motivational leaders (for which interactional centrality is important) can thus also have a social and external leadership role. In line with this point, further analyses reveal that, on average, 10.1% of the social leaders are also perceived as best task leaders, whereas 11.7% are also perceived as best motivational leaders. For external leaders, these percentages are respectively 9.8% and 10.2%. These percentages were very similar across the different sports. Although this reasoning also holds for other sports, it should be noted that for soccer, volleyball, and water polo the link between motivational leadership and interactional centrality was also significant. In these sports it is thus more likely that off-field leaders will also occupy an on-field leadership role (i.e., either task or motivational) for which interactional centrality is important.

We also investigated the importance of interactional centrality for the team captain—the formal leader of the team who can have either an on-field leadership role, an off-field leadership role, or no leadership role at all (for details, see [17]). In six out of the eight sports (the exceptions being basketball and ice hockey), team captains were more likely to occupy a central playing position than other team members. This pattern corroborates the findings of previous research that has identified a link between captaincy and interactional centrality [12–14]. Nevertheless, the exceptions of basketball and ice hockey are also in line with our expectations in so far as in these sports it is not necessary for the leader to occupy a central position to be able to communicate with all other team members and strategically influence the game. These findings are also consistent with, and allow us to make sense of, the non-significant findings of Roy [16], who studied the relation between interactional centrality and leadership in ice hockey.

Overall, we can thus conclude that, in line with H1, interactional centrality is closely linked to on-field leadership and captaincy, whereas its link with off-field leadership is weaker. As anticipated, in sports characterized by position switching during the game, the impact of interactional centrality was not as strong as it was in sports with larger fields and relatively fixed positions. Testifying to the generalizability of these patterns, it is also apparent that, with some exceptions, our findings were highly consistent for male and female teams and across a range of performance levels.

### The Relationship between Interactional Centrality and Leadership Quality

Although many previous studies have investigated whether leaders more often occupy a central position than non-leaders, none of these studies have determined whether players in central positions are also perceived as better leaders than their counterparts in non-central positions. In other words, besides being linked with occupation of a leader role, is interactional centrality also linked with leadership *quality*? Findings from Studies 3 and 4 provide a definitive answer to this question in showing that players who occupy a central field position were indeed perceived as better leaders in general, better task leaders, and better external leaders than those who had peripheral positions. Study 3 also revealed significant differences with respect to motivational leadership, but these were not replicated in Study 4. Only in the case of the social leadership role was the centrality of a player’s position not positively related to his/her perceived leadership quality.

The results regarding general leadership were consistent across male and female athletes, teams playing at high and at low performance levels, and in all of the examined sports. Only one exception emerged: in basketball, no significant differences were found between the leadership quality of central and non-central players. However, given that the communication opportunities between basketball players are very similar regardless of the centrality of an athlete’s playing position (in contrast, say, to soccer) it makes sense that here interactional centrality in unrelated to leadership quality.

Examination of the variability in the strength of the association between leadership quality and interactional centrality across male and female teams, performance levels, and various sports produced less clear-cut findings. First, the findings of Study 3 indicated that the relationship between interactional centrality and leadership quality was especially apparent in male teams, in high-level teams, and in soccer teams. In basketball and volleyball, no differences in leadership quality emerged between central and non-central players, which can again be explained by the fact that equivalent opportunities for communication are afforded to all team members. However, Study 4 revealed no differences across the different sports. Moreover, in the case of performance level, the findings of Study 3 were contradicted in that the link between interactional centrality and leadership quality was most evident in low-level, rather than in high-level teams. All in all, these results are rather mixed and suggest that player sex, level, and sport do not have a reliable bearing on the relationship between interactional centrality and leadership quality.

### Does Interactional Centrality Lead to Leadership or Does Leadership Lead to Interactional Centrality?

The results discussed above indicate that interactionally central positions tend to be occupied by those who occupy leadership roles, and who are also seen as good leaders by their teammates. Yet, because of its cross-sectional design, the present study provides limited insight into the causal underpinnings of this association. Is it the case that better leaders are given more central positions on the field? Or does this association result from the fact that players who are given central positions have more opportunities to interact with their fellow team members and hence to become better leaders?

One could argue that in some sports, a central position offers unique opportunities to structure the game. In volleyball, for example, the setter can decide which player will attack based on their own team’s or the opponent’s game strategy. In basketball too, the guard decides which strategy to adopt on the field. Likewise, in rugby the half backs have a key role to play in determining the speed and direction of attack through their ball distribution. Accordingly, it is to be expected that in such sports, task leaders—players who are skilled in making relevant decisions—will be chosen to occupy these central positions. However, one could also argue that the particular characteristics and experiences afforded by central positions furnish players with unique leadership development opportunities.

Another argument suggesting that ‘the position makes the leader’ is that in most sports players occupy specific positions throughout their career. This position choice is often made early in their career, based on a player’s technical and physical potential rather than on their leadership ability [36]. Furthermore, after transferring to a new club, players usually keep the same position, while their leadership status is heavily dependent on the particular team environment and thus often changes [18]. For example, a junior player might be an excellent leader in his junior team, but might not take the lead when being transferred to a senior team, even though he occupies the same position.

Balkundi, Kilduff, et al. [37] provided more insight into the causality of this relationship by examining advice networks of project teams in organizational settings. These advice networks are mapped by asking each of the team members to indicate the people from whom they seek advice about work-related matters. Given the focus on providing advice on task-related matters, these networks can be compared to the task leadership networks in sports teams. Here the researchers’ findings provided support for the causal influence of interactional centrality on leadership quality. More specifically, formal leaders who were initially central within the team advice network subsequently came to be seen as charismatic by their subordinates. In a sports setting, Grusky [7] corroborated these findings by studying a sample of professional baseball players and identifying the players that were later recruited to become managers. Findings revealed that players who had occupied central positions were more likely to become managers than players in peripheral positions.

Although all these studies suggest that central positioning is a basis for leadership development, this is clearly an issue for future experimental research to explore and resolve more conclusively. Nevertheless, from a theoretical standpoint (e.g., as suggested by [38]) we would hypothesize that this relationship is dynamic and reciprocal in nature—such that players who appear to be good leaders are more likely to be given central positions, but these positions in turn provide them with more opportunity to develop and prove their leadership potential. Again, though, there is clearly a need for future experimental and/or longitudinal research to investigate and resolve this question.

### Strengths, Limitations, and Avenues for Further Research

The present article has moved beyond previous knowledge in many ways. Its strengths include the variety of samples used, the inclusion of both males and females, and teams playing at high, low, and youth levels, in eight different sports. This broad sampling allowed us to compare findings across team sex, performance level, and sport, in ways that previous research was not able to.

Furthermore, we used a large number of participants to examine our research questions, as well as a design that improved upon previous research in a number of important ways. First, we did not only identify the position of the team captain, but also investigated the importance of interactional centrality for athlete leadership based on the perceptions of other team members. Given that team captains are not always perceived to be the best leaders [17], this afforded more insight into the link between interactional centrality and both formal and informal leadership. Second, we examined this relationship with regard to the four key roles that leaders can occupy (i.e., as task, motivational, social, external leader), thereby providing more insight in the importance of interactional centrality for both on-field and off-field leaders. Third, in addition to linking interactional centrality with leader appointment, we also demonstrated that a central position improved the perceived leadership quality of leaders and players.

Despite these strengths, some limitations should be acknowledged. First, we were unable to control for personal characteristics that potentially influence a player’s position, such as age, height, weight, speed, agility, or technical and tactical abilities. For example, previous studies have found clear differences in anthropometric and physiological characteristics between the different playing positions [36, 39]. More particularly, because age influences the physiological abilities of a player and at the same time is a characteristic attribute of leaders [19, 40], one might argue that age confounds the observed relationship between interactional centrality and leadership. For example, as they get older, soccer players may move from attacking positions to midfielder positions, given that these positions require less explosive power [36]. However, the experience they have gained over the years may also have increased their leadership potential. To explore whether age was indeed a confounding factor in the present case, we compared the age of players in central positions with those in non-central positions for each of the eight sports that we studied. Analyses revealed that in none of the studies did players’ playing position vary as a function their age, suggesting that this was not a contributory factor in the patterns we have identified. Nevertheless, an interesting avenue for future research would be to determine whether (and how) other personal characteristics or technical and tactical abilities influence the relationship between interactional centrality and leadership.

A second limitation of the present research is that it sheds no light on the causal mechanisms that underlie the link between interactional centrality and leadership quality. We have suggested that the observed leadership quality of central players is a consequence of the more intense communication and enhanced opportunities to tactically influence the game strategy that these positions afford. Nevertheless, we are not in a position to confirm that this is the case. In this regard, previous research has highlighted the role of communication in fostering team members’ identification with their group, in ways that help to create a shared sense of ‘us’ [41] and it would be interesting to observe whether these same processes are implicated in the patterns reported above. Here we would suggest that the increased communication opportunities, inherent to a central position, give leaders greater scope to engage in identity leadership by creating, representing, and advancing a shared sense of ‘us’ on the field and that this in turn results in a higher perceived leadership quality [42]. This is a possibility that we are exploring in follow-up studies that are currently underway. Clearly, though, future research could identify a range of other mechanisms that might underlie the link between interactional centrality and leadership quality.

## Conclusions

The findings of the present studies indicate that the position players occupy on a sporting field is highly predictive of their capacity to fulfil a leadership role. More specifically, players who have positions that are interactional central are more likely to display on-field leadership of both a task and a motivational form. This relationship is attenuated in sports where an interactionally central position confers limited interactional advantages but, beyond this, the pattern is broadly consistent across different sports, different sexes, and different levels of skill. These patterns are consistent with the idea that interactionally central positions afford players with greater opportunities to *do* leadership—either through communication or through action. Significantly too, they also provide a basis for them to *be seen to do* leadership by others on their team. Thus while it is often stated that “leadership is an action, not a position” [43], it is nevertheless the case that, when it comes to performing that action, some positions are clearly more advantageous than others.

## Supporting Information

S1 Table The playing positions of appointed athlete leaders and the team captain in the different sports (Study 1). (DOCX)

S2 Table The valid percentage of male and female athlete leaders playing on a central playing position within each of the examined sports (Study 1). (DOCX)

S3 Table The valid percentage of high-level, low-level, and youth athlete leaders playing in a central playing position within each of the examined sports (Study 1). (DOCX)
