# Supplementary material for: Leading from the Centre: A Comprehensive Examination of the Relationship between Central Playing Positions and Leadership in Sport
Source: PLoS One. 2016 Dec 15;11(12):e0168150. doi: 10.1371/journal.pone.0168150 (PMC5158024; doi:10.1371/journal.pone.0168150)
Supplement: S1 Table — (DOCX) [file pone.0168150.s001.docx]

**S1 Table. The playing positions of appointed athlete leaders and the team captain in the different sports (Study 1).**

| Sport | Number of on-field positions | Position TL | Position ML | Position SL | Position EL | Position captain |
| --- | --- | --- | --- | --- | --- | --- |
| Basketball | Total: 5 |  |  |  |  |  |
| Guard^*^ | 2 | 676  (34.5%) | 279  (14.2%) | 204 (10.4%) | 151  (7.7%) | 484 (24.7%) |
| Forward | 2 | 298  (15.2%) | 427  (21.8%) | 389 (19.9%) | 199  (10.2%) | 403 (20.6%) |
| Center | 1 | 256  (13.1%) | 383  (19.6%) | 397 (20.3%) | 200  (10.2%) | 357 (18.2%) |
| *Multiple positions*^a^ |  | 206  (10.5%) | 188  (9.6%) | 131  (6.7%) | 73  (3.7%) | 209 (10.7%) |
| *Missing values*^b^ |  | 523  (26.7%) | 682  (34.8%) | 838 (42.8%) | 1,336 (68.2%) | 506 (25.8%) |
| Handball | Total: 7 |  |  |  |  |  |
| Wingman | 2 | 4  (3.4%) | 16  (13.8%) | 18  (15.5%) | 12  (10.3%) | 18  (15.5%) |
| Left/Right backcourt | 2 | 17  (14.7%) | 14  (12.1%) | 13  (11.2%) | 10  (8.6%) | 12  (10.3%) |
| Center backcourt^*^ | 1 | 54  (46.6%) | 8  (6.9%) | 8  (6.9%) | 3  (2.6%) | 16  (13.8%) |
| Pivot | 1 | 8  (3.9%) | 16  (13.8%) | 10  (8.6%) | 7  (6.0%) | 13  (11.2%) |
| Goal keeper | 1 | 2  (1.7%) | 9  (7.8%) | 11  (9.5%) | 6  (5.2%) | 10  (8.6%) |
| *Multiple positions*^a^ |  | 13  (11.2%) | 11  (9.5%) | 4  (3.4%) | 1  (0.9%) | 7  (6.0%) |
| *Missing values*^b^ |  | 18  (15.5%) | 42  (36.2%) | 52  (44.8%) | 77  (66.4%) | 40  (34.5%) |
| Soccer | Total: 11 |  |  |  |  |  |
| Goal keeper | 1 | 20  (3.4%) | 31  (5.3%) | 27  (4.6%) | 10  (1.7%) | 28  (4.8%) |
| Back defender | 2 | 20  (3.4%) | 26  (4.4%) | 34  (5.8%) | 15  (2.5%) | 37  (6.3%) |
| Central defender^*^ | 2 | 111  (18.8%) | 78  (13.2%) | 47  (8.0%) | 29  (4.9%) | 110  (18.7%) |
| Wing | 2 | 10  (1.7%) | 26  (4.4%) | 28  (4.8%) | 9  (1.5%) | 22  (3.7%) |
| Midfielder^*^ | 2 | 220  (37.4%) | 147  (25%) | 104  (17.7%) | 52  (8.8%) | 201  (34.1%) |
| Forward | 2 | 27  (4.8%) | 28  (4.8%) | 29  (4.9%) | 6  (1.0%) | 25  (4.2%) |
| *Multiple positions*^a^ |  | 51  (8.7%) | 49  (8.3%) | 51  (8.7%) | 27  (4.6%) | 33  (5.6%) |
| *Missing values*^b^ |  | 129  (21.9%) | 204  (34.6%) | 269  (45.7%) | 4  (0.7%) | 133  (22.5%) |
| Volleyball | Total: 7 |  |  |  |  |  |
| Outside hitter | 2 | 325  (25.3%) | 254  (19.7%) | 198  (15.4%) | 147  (11.4%) | 757  (58.8%) |
| Opposite | 1 | 81  (6.3%) | 87  (6.8%) | 77  (6.0%) | 63  (4.9%) | 61  (4.7%) |
| Middle player | 2 | 145  (11.3%) | 180  (14%) | 199  (15.5%) | 128  (9.9%) | 181  (14.1%) |
| Setter^*^ | 1 | 253  (19.7%) | 181  (14.1%) | 160  (12.4%) | 123  (9.6%) | 225  (17.5%) |
| Libero | 1 | 68  (5.3%) | 81  (6.3%) | 51  (4.0%) | 38  (3.0%) | 10  (0.8%) |
| *Multiple positions*^a^ |  | 97  (7.5%) | 35  (2.7%) | 76  (5.9%) | 37  (2.8%) | 51  (4.0%) |
| *Missing values*^b^ |  | 318  (24.7%) | 76  (5.9%) | 526  (40.9%) | 751  (58.4%) | 2  (0.2%) |
| Hockey | Total: 11 |  |  |  |  |  |
| Goal keeper | 1 | 8  (6.3%) | 5  (3.9%) | 15  (11.8%) | 4  (3.1%) | 3  (2.4%) |
| Defender | 4 | 41  (32.3%) | 25  (19.7%) | 32  (25.2%) | 22  (17.3%) | 39  (30.7%) |
| Midfielder^*^ | 3 | 37  (29.1%) | 31  (24.4%) | 22  (17.3%) | 21  (16.5%) | 39  (30.7%) |
| Forward | 3 | 7  (5.5%) | 13  (10.2%) | 11  (8.7%) | 8  (6.3%) | 12  (9.4%) |
| *Multiple positions*^a^ |  | 7  (5.5%) | 7  (5.5%) | 1  (0.8%) | 4  (3.1%) | 5  (3.9%) |
| *Missing values*^b^ |  | 27  (21.3%) | 46  (36.2%) | 46  (36.2%) | 68  (53.5%) | 29  (22.9%) |
| Ice Hockey | Total: 6 |  |  |  |  |  |
| Goal keeper | 1 | 3  (4.2%) | 3  (4.2%) | 6  (8.3%) | 5  (6.9%) | 1  (1.4%) |
| Defender | 2 | 22  (30.6%) | 16  (22.2%) | 14  (19.4%) | 7  (9.7%) | 29  (40.3%) |
| Attacker | 2 | 17  (23.6%) | 17  (23.6%) | 11  (15.3%) | 5  (6.9%) | 23  (31.9%) |
| Center^*^ | 1 | 16  (22.2%) | 11  (15.3%) | 4  (5.6%) | 2  (2.8%) | 12  (16.7%) |
| *Multiple positions*^a^ |  | 4  (5.6%) | 2  (2.8%) | 4  (5.6%) | 2  (2.8%) | 2  (2.8%) |
| *Missing values*^b^ |  | 10  (13.9%) | 23  (31.9%) | 33  (45.8%) | 51  (70.8%) | 5  (6.9%) |
| Rugby | Total: 15 |  |  |  |  |  |
| Prop | 2 | 8  (9.5%) | 4  (4.8%) | 7  (8.3%) | 2  (2.4%) | 9  (10.7%) |
| Hooker | 1 | 2  (2.4%) | 0 | 1  (1.2%) | 7  (8.3%) | 3  (3.6%) |
| Lock | 2 | 3  (3.6%) | 8  (9.5%) | 5  (6.0%) | 1  (1.2%) | 1  (1.2%) |
| Flanker | 2 | 10  (11.9%) | 8  (9.5%) | 3  (3.6%) | 2  (2.4%) | 6  (7.1%) |
| Scrum-half^*^ | 1 | 2  (2.4%) | 5  (6.0%) | 1  (1.2%) | 0  (0 %) | 0  (0 %) |
| Fly-half^*^ | 1 | 17  (20.2%) | 8  (9.5%) | 2  (2.4%) | 3  (3.6%) | 15  (17.9%) |
| Centre | 2 | 12  (14.3%) | 8  (9.5%) | 1  (1.2%) | 2  (2.4%) | 12  (14.3%) |
| Winger | 2 | 1  (1.2%) | 1  (1.2%) | 11  (13.1%) | 7  (8.3%) | 1  (1.2%) |
| Full-back | 1 | 2  (2.4%) | 1  (1.2%) | 1  (1.2%) | 2  (2.4%) | 3  (3.6%) |
| Nr. 8^*^ | 1 | 10  (11.9%) | 9  (10.7%) | 4  (4.8%) | 3  (3.6%) | 11  (13.1%) |
| *Multiple positions*^a^ |  | 7  (8.3%) | 5  (6.0%) | 7  (8.3%) | 6  (7.1%) | 7  (8.3%) |
| *Missing values*^b^ |  | 10  (11.9%) | 27  (32.1%) | 41  (48.9%) | 47  (56.0%) | 16  (19.0%) |
| Water polo | Total: 7 |  |  |  |  |  |
| Goal keeper | 1 | 1  (1.0%) | 7  (7.1%) | 5  (5.1%) | 0  (0 %) | 0  (0 %) |
| Point^*^ | 1 | 32  (32.3%) | 15  (15.2%) | 12  (12.1%) | 9  (9.1%) | 26  (26.3%) |
| Flat | 2 | 4  (4.0%) | 1  (1.0%) | 2  (2.0%) | 2  (2.0%) | 4  (4.0%) |
| Wing | 2 | 5  (5.1%) | 6  (6.1%) | 8  (8.1%) | 2  (2.0%) | 9  (9.1%) |
| Center | 1 | 19  (19.2%) | 13  (13.1%) | 9  (9.1%) | 6  (6.1%) | 12  (12.1%) |
| *Multiple positions*^a^ |  | 21  (21.2%) | 15  (15.2%) | 13  (13.1%) | 6  (6.1%) | 15  (15.2%) |
| *Missing values*^b^ |  | 17  (17.2%) | 42  (42.4%) | 50  (50.5%) | 74  (74.7%) | 33  (33.3%) |

The positions indicated by an asterisk have been defined as central playing positions.

TL = task leader; ML = motivational leader; SL = social leader; EL = external leader

^a^ Players who indicated multiple positions for the appointed leader.

^b^ Missing values arose because either (1) the particular leader was not present in the team; (2) participants did not fill out the position of the leader; or (3) the position that was filled out did not match the defined categories.
