# Supplementary material for: Leading from the Centre: A Comprehensive Examination of the Relationship between Central Playing Positions and Leadership in Sport
Source: PLoS One. 2016 Dec 15;11(12):e0168150. doi: 10.1371/journal.pone.0168150 (PMC5158024; doi:10.1371/journal.pone.0168150)
Supplement: S2 Table — (DOCX) [file pone.0168150.s002.docx]

**S2 Table. The valid percentage of male and female athlete leaders playing on a central playing position within each of the examined sports (Study 1).**

|  |  | Task leader | | Motivational leader | | Social leader | | External leader | | Team captain | |
| --- | --- | --- | --- | --- | --- | --- | --- | --- | --- | --- | --- |
| Leaders’ sex |  | Male | Female | Male | Female | Male | Female | Male | Female | Male | Female |
| Total sample |  | 52.2 | 42.9 | 34.8 | 65.2 | 29.1 | 19.8 | 31.9 | 25.7 | 26.5 | 39.3 |
| Sport-specific | Reference percentage |  |  |  |  |  |  |  |  |  |  |
| Basketball | 40.0 | 52.2^***^ | 61.0^***^ | 28.3 | 20.0 | 23.3 | 15.3 | 26.9 | 28.4 | 38.6 | 39.6 |
|  |  |  |  |  |  |  |  |  |  |  |  |
| Soccer | 36.4 | 82.1^***^ | 67.6^***^ | 67.3^***^ | 63.9^***^ | 54.9^***^ | 68.0^***^ | 66.3^***^ | 70.6^**^ | 74.0^***^ | 69.2^***^ |
|  |  |  |  |  |  |  |  |  |  |  |  |
| Volleyball | 14.3 | 27.7^***^ | 27.5^***^ | 22.5^***^ | 21.8^***^ | 25.2^***^ | 20.4^***^ | 29.2^***^ | 19.8^**^ | 22.0^***^ | 15.6 |
|  |  |  |  |  |  |  |  |  |  | (χ² = 8.19; *p* < .01) | |
| Handball | 14.3 | 62.3^***^ | 66.7^***^ | 17.1 | 4.5 | 17.1 | 5.3 | 12.0 | / | 27.7^**^ | 13.6 |
|  |  |  |  |  |  |  |  |  |  | *ns* | |
| Hockey | 27.3 | 31.4 | 50.0^**^ | 29.3 | 57.6^***^ | 22.2 | 34.3 | 20.6 | 66.7^***^ | 40.4^*^ | 43.9^*^ |
|  |  | *ns* | | (χ² = 5.93; *p* < .05) | |  |  | (χ² = 11.78; *p* < .001) | |  |  |
| Ice hockey | 16.7 | 29.4^*^ | / | 25.6 | / | 12.5 | / | 11.1 | / | 21.1 | / |
|  |  |  | |  |  |  |  |  |  |  |  |
| Rugby | 20.0 | 47.2^***^ | 28.6^*^ | 42.2^***^ | / | 21.2 | / | 20.0 | 22.2 | 52.1^***^ | 7.7 |
|  |  |  | |  | |  |  |  |  | (χ² = 8.11; *p* < .01) | |
| Water polo | 14.3 | 57.4^***^ | / | 42.9^***^ | / | 40.7^**^ | / | 50.0^***^ | / | 57.8^***^ | / |

^*^*p* < .05; ^**^*p* < .01; ^***^*p* < .001 (based on a *z*-test, comparing the percentage against the reference percentage)

*Notes:* The significance levels indicate the percentages which are significantly higher than the reference percentage for that particular sport.

The percentages of the groups containing less than 10 valid cases have been omitted.

If the comparison between the percentage of leaders in a central position and the reference percentage differs for male and female leaders (i.e., for males it is significantly higher and for females it is not or vice-versa), the ‘N-1’ Chi-squared test indicates whether the difference between male and female leaders is significant.
